# Intertwining Density Functional Theory and Experiments in the Investigation of Gas Sensing Mechanisms: A Review

**DOI:** 10.3390/s25030867

**Published:** 2025-01-31

**Authors:** Paulina Powroźnik, Maciej Krzywiecki

**Affiliations:** Institute of Physics—Center for Science and Education, Silesian University of Technology, 44-100 Gliwice, Poland; maciej.krzywiecki@polsl.pl

**Keywords:** gas sensors, sensing mechanisms, DFT, quantum chemistry, experiment, theoretical modeling

## Abstract

In this review, we present the last ten years of progress in evaluation of gas sensing mechanisms. We focus mostly on the studies joining theoretical modeling of gas adsorption by density functional theory method with advanced experimental characterization of sensing materials. We provide the background about important aspects that should be taken into account during the design of the effective sensing device and an overview of the most recently studied sensing materials and analytes. Using the exemplary works, we next show how theory and experiment intertwine in revealing how the sensing mechanism serves to improve the device performance. In the end, we summarize the progress already made despite the existing difficulties, and provide an outlook for future methodological development.

## 1. Introduction

**Figure 1:**
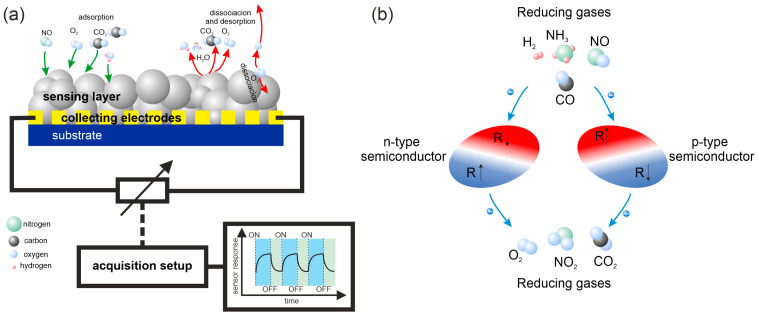
(**a**) The general principle of gas sensor operation. (**b**) The changes in electrical resistance (R) caused by electron transfer during the adsorption of oxidizing or reducing gases on n-type and *p*-type semiconductors.

Gas sensors are electronic devices in which operation is based on the chemical or physical interaction of the surface of sensitive material with gas molecules present in the surrounding atmosphere. Such interaction influences some physical parameters of the material. The change in these parameters is next transformed into an electronic signal, i.e., sensor response. The general principle of gas sensor operation is presented in Figure 1a. The physical parameter of the materials that is most commonly utilized in gas sensors is electrical conductivity. Gas adsorption on the surface of the sensing structure causes electronic charge transfer or reorganization, which leads to the alteration in the electronic structure and, as a consequence, changes in the surface conductivity and the electrical resistance of the device. Depending on the sensing material-gas combination, the resistance either increases or decreases. For n-type semiconductors, adsorption of oxidizing gases causes an increase in resistance while after interaction with reducing gases the resistance decreases. For *p*-type materials, the effect is the opposite (Figure 1b). However, several other material properties can be transduced into a sensor response, e. g., electronic work function, photoluminescence, and thermal properties. The common part of the operation of all kinds of the aforementioned gas sensors is the adsorption of gas molecules on the sensitive materials and the resulting alteration of the material properties. How this interaction occurs and how it influences the device is referred to as the gas sensing mechanism. The proper utilization of sensing devices requires an in-depth recognition of this mechanism.

Researchers have been struggling to overcome difficulties in designing gas sensors for many years. Most commonly used materials as gas-sensitive layers are semiconductive metal oxides (SMOs). However, their greatest limitations are high working temperature and poor selectivity. There are many ways to improve sensing device parameters, such as the application of SMO nanostructures [1,2,3,4], doping of SMOs with metals [5,6,7,8,9], formation of heterostructures [10,11,12,13], or replacement of inorganic SMOs with organic materials [14,15,16,17]. Given the number of various possibilities for improvements, the investigation of new sensing structures should be somehow structured in a way to achieve the highest possible efficiency in the sensor device. It is especially important nowadays due to the greater awareness of the urgent need for monitoring substances that pose a threat to both the environment and public health.

Fortunately, with the extensive progress in both experimental surface science and computational quantum chemistry methods for materials parameters modeling, scientists have tools to comprehensively study sensing materials and understand the mechanisms of gas interaction with surfaces. This leads to the possibility of increased awareness in designing devices with the desired parameters, primarily focused on selectivity improvement.

This review summarizes the progress already made in intertwining the theory and experiment for investigating gas sensing mechanisms. We present the methods applied in both—theoretical modeling of gas adsorption and experimental studies of actual sensing structures—aiming to provide methodological hints for future studies. Due to the large number of reports regarding gas sensing mechanisms published in the last few years, we limited our research to the works that join theory and experiments, resulting in a comprehensive description of the sensing mechanism. We show which combinations of sensing material–gas have already been studied, which experimental and theoretical methods have been applied, and which properties can be compared between experimental and theoretical results, thus giving consistency in the established mechanism.

## 2. Objects of the Investigation

### 2.1. Studied Analytes

Most of the research on gas sensors in the last several years has focused on four groups of analytes, which can be distinguished as follows:

I. Volatile organic compounds (e.g., ammonia, acetone, ethanol, methanol, trimethylamine, chloroform)
II. Atmosphere pollutants (e.g., NO_x_, CO_2_, O_3_, HCl, H_2_S)
III. Explosive gases (e.g., H_2_, methane)
IV. Gases resulting from electrical equipment failures (e.g., H_2_S, SOF_2_, SO_2_, SO_2_F_2_)

Group I can be considered in two aspects. VOCs can be released into the atmosphere during industrial and laboratory processes or from products such as cosmetics, detergents, and medicines. Their high concentrations are toxic, posing a threat to human health. On the other hand, low concentrations of VOCs in human breath are markers of some metabolic diseases, such as diabetes, liver failure, etc. Group II contains compounds resulting from human activity that in high concentration in the atmosphere are a threat to the environment and to human health. Many studies in the last several years have focused on the fast detection of low H_2_ concentrations. It became very important due to the application of hydrogen in industry, but also as a potential energy source for vehicles. As hydrogen is an explosive gas, it is crucial to be able to detect even the smallest leakages. The last analyte group we distinguished is gases resulting from the failures in electrical equipment (e.g., insulation in transformers). The detection of these gases can serve a double scope—diagnosis of the failures and human protection from the toxic substances.

The aforementioned groups of analytes together with materials studied for their detection are summarized in Table 1.

### 2.2. Studied Sensing Materials

In the past, the most studied materials for gas sensors were SMOs, mostly tin dioxide, which is commonly applied in commercial devices. The reasons for using these kinds of materials were their sensitivity to a broad range of analytes, good chemical and thermal stability, and the possibility for relatively easy deposition of layers. However, with developing science, technology, and environmental awareness, the need for selectively sensing a large variety of gases has grown. Pristine SMO films lack selectivity. Also, devices based on SMOs require high working temperatures, which imply high power consumption. Nowadays, all efforts in materials science are focused on finding more unified economical and environmentally friendly solutions. For these reasons, gas sensor research aims to develop selective sensors that work at lower temperatures than typical SMO devices—at room temperature in the best scenario.

The most of works combining experiments and theory in the last several years aim to find a way to improve the performance of SMOs as gas sensing materials. Zinc oxide became a popular material recently [7,8,10,13,18,19,20,21]. It is often combined with other sensitive materials in heterojunctions [10,13,18]. Another method for obtaining better sensing results is doping with noble or transition metals [7,8,21] or creating ZnO nanostructures [20]. The ways to improve the performance of metal oxides are generally common for all SMOs, not only ZnO. Other metal oxides that were studied in the last several years are CuO [10,11,22], TiO_2_ [1,2], NiO [11,23], MoO_x_ [24,25], In_x_O_y_ [6,12], WO_3_ [3,26], Fe_2_O_3_ [27,28], CeO_2_ [5], and Co_3_O_4_ [29]. Even though SnO_2_ has been the SMO most applied in gas sensing devices for more than 50 years, it is still being studied to improve its parameters [4,11,27,28,29,30,31,32,33,34,35]. Apart from these methods for boosting the capability of SMOs, several studies presented the scheme for gas adsorption controlled through the oxide’s layer stoichiometry, mostly by introducing oxygen vacancies [23,24,25].

Another large group of materials investigated as sensitive layers are organic semiconductors [14,15,16,17,36,37,38,39] and graphene [31,40,41,42]. They are usually functionalized, similarly to SMOs, either by forming the heterojunction [31] or by adding functional groups as well as modifying with noble or transition metals [17,40]. Examples of gas-sensitive organic semiconductors are polymide [14], squariaine [42], various frameworks and polymers [15,16,17,36,39], tellurene [38], and liquid crystals [43].

Finally, the last group is inorganic compounds that are not oxides, such as SnS [44], SnSe_2_ [34,45], MXene [46], Co_12_ cluster [18], WS_2_ [47], and MoS_2_ [47].

Table 1 summarizes the materials applied in the investigation of gas sensors that join experiments with theory. For each analyte, we show sensing structures that were studied for selective detection and the respective operating temperatures.

**Table 1:** Summary of materials applied in studies of selective gas sensors that join experiments with theory.

Analyte	Sensing Structures	Operating Temperature
O_3_	CuO (111) surface [22], chematite microrhombuses [48]	250 °C [22], 150 °C [48]
H_2_S (hydrogen sulfite)	Au-modified graphene [40], 3D flower-like Ni-doped CeO_2_ [5], NiO with oxygen vacancies [23], defect-enriched MoO_3_ [24], Co_12_ and ZnO/Co_12_ [18]	Room temperature [40], 200 °C [5], 120–210 °C [23], 140 °C [24], 115 °C [18]
SOF_2_	Au-modified graphene [40]	Room temperature [40]
HCl (hydrochloric acid)	porphyrinated polyimide honeycomb films [14]	From room temperature to 300 °C [14]
NH_3_ (ammonia)	graphene [41], covalent triazine-based frameworks [15], coral-like Au-SnSe_2_ [45], PEDOT:PSS [36], ZnO/CuO heterojunction [10], nitrogen-contained covalent organic polymers and frameworks [16], SnO_2_ nanoparticles-modified a-Fe_2_O_3_ [28]	Room temperature [10,15,16,27,36,41,45]
CO_2_ (carbon dioxide)	squariaine [37], amine-functionalized ZIF-8 with 3-amino-1,2,4-triazole [17]	Room temperature [17,37]
NO (nitrogen oxide)	ZnO thin films [19], tri-graphene [42]	200 °C [19], Room temperature [42]
O_2_ (oxygen)	nano-sized black TiO_2_ [1]	Room temperature [1]
CO (carbon monoxide)	hybrid NiO/TiO_2_ nanofiber [2], co-doped In_2_O_3_/MoSe_2_ [6], PtAg-modified WO_3_ nanorods [3], cir-graphene [42], CuO-SnO_2_ [11]	Room temperature [2,3,6,42], 20 °C [11]
C_3_H_6_O (acetone)	SnO_2_ thick film [1], WO_3_ [26]	180 °C [1], 300 °C [26]
SF_6_	Ni-doped ZnO (100) surface [7], SnO_2_-graphene [31]	200–300 °C [31]
N(CH₃)₃ trimethylamine	oxygen-vacancy-enriched porous α-MoO_3_ nanosheets [25]	133 °C [25]
H_2_ (hydrogen)	Pt-decorated ZnO nanotube [20], Ag_9_Pt/ZnO(1010) surface [49], Pd_4_ cluster-decorated SnO_2_ [32], doped SnO_2_ [33]	Room temperature [20], 200 °C [32]
C_6_H_5_NH_2_ (aniline)	Pd-doped ultrathin agaric-like ZnO [8]	280 °C [8]
NO_2_ (nitrogen dioxide)	tellurene nanoflake [38], atomically dispersed Fe [49], ionic-conjugation covalent organic frameworks [39], nitrogen-contained covalent organic polymers and frameworks SnO2/SnSe2 heterostructures [34], MoSe_2_/Co_3_O_4_ [50], NiO/In_2_O_3_ [12], quantum dots-sensitized MXene [46], MoS_2_/WS_2_ nanoflowers [51], metal ion-substituted ZnFe_2_O_4_ core-shell microspheres [28]	Room temperature [12,16,34,38,46,49,50,51], 80 °C [39], 120 °C [28]
CH₃OH (methanol)	Zn-substituted SnS [44]	Room temperature [44]
C₂H₆O (ethanol)	Sr-doped cubic In_2_O_3_/rhombohedral In_2_O_3_ homojunction [9], Co3O4(110) surface [29]	300 °C [9], 150 °C [29]
CHCl₃ (chloroform)	SnO_2_ nanostructured thin film [4]	Room temperature [4]
C_2_H_2_ (acetylene)	Pd_4_ cluster decorated SnO_2_ [32]	200 °C [32]
CH₄ (methane)	ZnO with dispersed Pd [21]	230 °C [21]
2-C_3_H_7_OH (2-propanol)	Al2O3/ZnO heterostructure [13]	350 °C [13]
n-C_4_H_9_OH (n-butanol)	Al2O3/ZnO heterostructure [13]	350 °C [13]
C_6_H_15_NO_3_ (triethanolamine)	Hydrogen-bonded liquid crystals [43]	Room temperature [43]

## 3. Theoretical Methods

Since the sensing mechanisms are mainly ruled by the physiochemical processes occurring at the material’s surface during the gas adsorption, computational quantum chemistry methods become useful in studying at the theoretical level the behavior of the potential sensor. The most efficient group of methods for studying the interaction of molecules with surfaces is the one based on density functional theory (DFT). Many computational programs apply DFT formalism. However, few of them are commonly used in gas sensor investigations. The most popular packages are Vienna ab initio Simulation Package (VASP) [4,5,9,13,17,24,25,27,29,38,49,50,51] and DMol^3^ [7,10,30,31,32,40,42,45]. Other packages also used in gas sensing mechanisms studies are Gaussian [14,15,37,43], GAMESS [20,52,53], SIESTA [22,37], Quantum Espresso [19,54], CASTEP [34,39,41,44], and CRYSTAL [48]. The popularity of the DMol^3^ package is due to the efficient code for the adsorption geometry optimization—the crucial calculation for gas sensors—while VASP is one of the most commonly used programs with DFT implementation in general.

Regardless the applied software, there are several parameters that have to be carefully chosen for each DFT calculation. The fundamental one is the exchange–correlation (XC) functional. Additionally, various approximation methods for dispersion interactions can be included within the XC functional. Next, proper basis sets or pseudopotentials and cutoff energy have to be selected, depending on which type of basis sets are implemented within the code, based on atomic orbitals or plane wave basis sets, respectively. For the calculations with plane wave basis sets or basis sets based on the atomic orbital, which take into account periodic boundary conditions, the size of the unit cell and k-grid sampling also have to be preset. The combination of all these settings decides the accuracy and computational cost of the simulation. For this reason, the proper choice of input parameters is crucial and yet the most difficult part of any DFT calculation. The choice can be based on a benchmark calculation before the final one, comparing the results with experimental data, or a literature review for the analog systems. The scope of this section is to suggest the applied codes and input settings in DFT gas sensor research. Table 2 presents the XC functionals and basis set/pseudopotential combinations used for modeling gas sensing mechanisms, together with respective materials, applied software, and literature references. For more detailed information about the simulation setups, the reader is encouraged to search the respective references. As one can see from Table 2, the most popular functional is the Perdew–Burke–Ernzerhof (PBE) method of the generalized gradient approximation (GGA). It is occasionally expanded with dispersion interaction corrections (Grimme or Grimme 3) and/or the DFT + U framework for a more accurate electronic structure. Some studies apply the all-electron B3LYP functional. However, due to the higher computational cost of all-electron functionals, it is usually effective for systems without periodic boundary conditions such as organic molecules or metal oxide nanoclusters.

**Figure 2:**
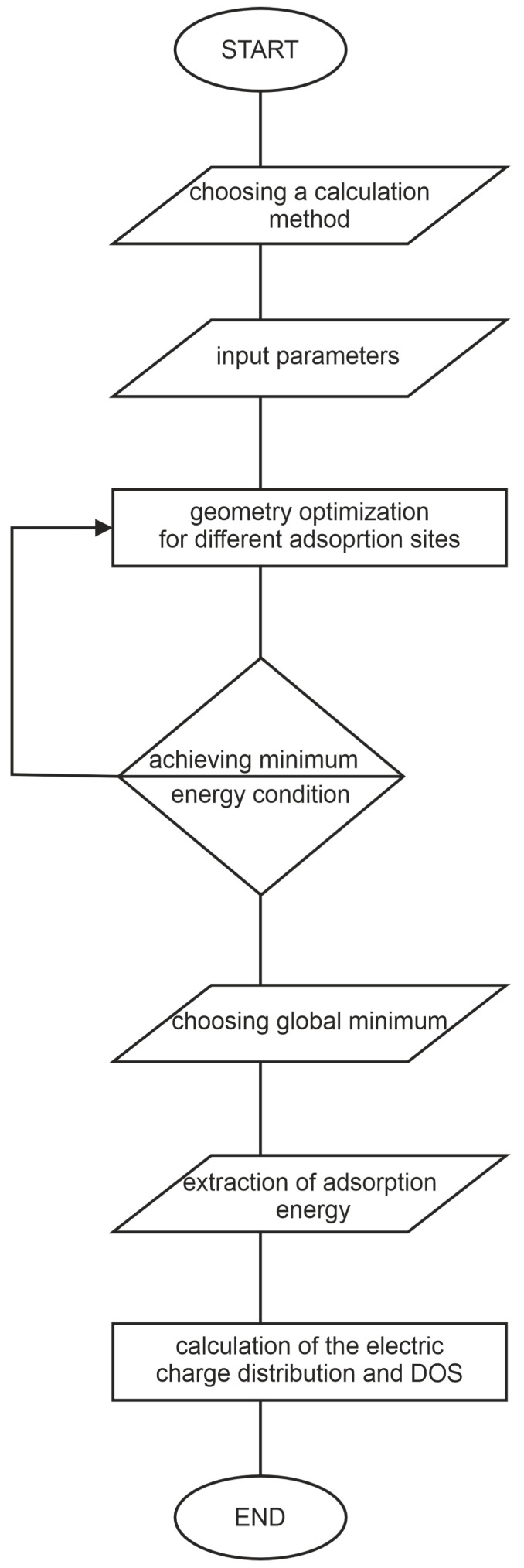
The scheme of the DFT calculation and output data extraction procedure.

After choosing the parameters for the simulation and running it, one needs to extract the relevant output data. For the gas sensing mechanism investigation, the crucial ones are adsorption geometry and energy—the higher the adsorption energy, the stronger the interaction of the gas with the sensing material’s surface. Thus, knowing the expected adsorption energy, one can predict if the considered analyte will adsorb on the chosen material’s surface by physisorption or chemisorption. This knowledge can help to design the device for specific applications. Physisorption or weak chemisorption is preferred when the sensor’s reusability is crucial, while strong chemisorption is better if one-time detection of very low concentrations of highly toxic gases is required. For finding the preferential adsorption sites and geometries, geometry optimization has to be run for different starting structures to find the global minimum (the final structure with the lowest total energy of formation). Next, for the chosen preferential adsorption geometries, the auxiliary data are often calculated, such as electronic charge distribution and density of states (DOS and PDOS). The electronic charge transfer/redistribution between the sensor’s material and the detected gas molecules is the basis for the sensor response acquisition in the case of chemo-resistive gas sensors, as shown in Figure 1. The DOS spectra, especially when compared with experimental photoemission study, provide a deeper insight into the mechanisms of charge redistribution and electronic sensitization. For the electronic charge distribution, Mulliken as well as Bader formalism can be applied, while DOS calculations are usually run with the same parameters as geometry optimization or sometimes with denser k-grid sampling for more accurate results. The scheme of the DFT calculation and output data extraction procedure is presented in Figure 2.

**Figure 3:**
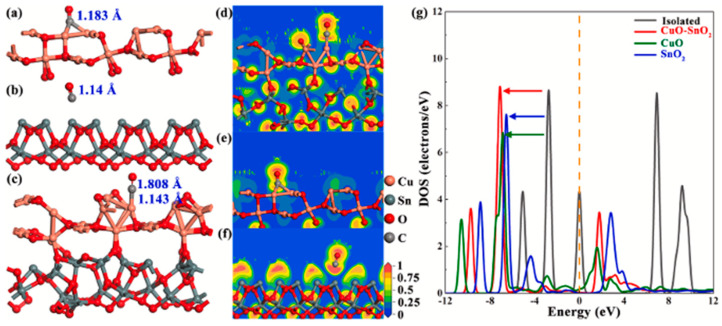
An example of DFT calculation results obtained by Weiqi Wang et al. in the study of CO CuO–SnO_2_ sensor as an illustration of representative outcomes of DFT modeling in gas sensing studies. Side views of the most stable adsorption geometries for the (**a**) CuO_(1)__CO, (**b**) SnO_2(1)__CO, and (**c**) CuO_(1)–(1)_SnO2___CO systems, respectively. Charge distribution for the (**d**) CuO_(1)_–SnO_2(1)__CO, (**e**) CuO_(1)__CO, and (**f**) SnO_2(1)__CO systems, respectively. (**g**) DOS of isolated and adsorbed CO molecules. Reproduced from [11] with permission from Elsevier.

Figure 3 shows an example of the most stable adsorption geometries and corresponding charge transfer distribution together with DOS spectra from the work of Weiqi Wang et al. [11] regarding the CuO–SnO_2_ sensor for room-temperature CO detection. The analysis of adsorption geometries of CO molecules on three kinds of substrates (CuO, SnO_2_, and CuO-SnO_2_) showed that elongation of the C-O bond occurs after the adsorption of the molecules on sensing materials, signifying the activation behavior of the CO molecule after the interaction. Further exploration of interatomic distances between CO molecules and substrates together with corresponding charge distribution suggested that physisorption is the dominating sensing mechanism. The shifts of the peaks from the CO molecule in DOS spectra after adsorption confirmed the interaction between the molecular orbital of CO and the sensing materials.

## 4. Experimental Methods

Theoretical modeling, as shown in the previous subsection, can provide insight into the sensing mechanisms for any chosen system on the atomic level. However, to design an effective device working in real conditions, an experimental investigation is crucial. The process of experimental studies of new gas sensing systems can be generally divided into two stages: fundamental surface characterization and gas sensing performance testing.

For the first stage, the advanced methods of surface science are employed. The experiments that are usually performed are summarized in Table 3 together with the information useful for gas sensors that can be obtained. In general, in terms of information about the sensing layer and its surface, some groups of methods can be distinguished. The structural and chemical characterization is performed using X-ray diffraction, Raman spectroscopy, nuclear magnetic resonance spectroscopy, and photoemission spectroscopies. For the morphological characterization, various scanning microscopies (i.e., atomic force microscopy, scanning, and transmission electron microscopy) are applied. Sometimes Brunauer–Emmett–Teller (BET) analysis is used to determine the specific surface area. From these methods, important information about adsorption site availability is obtained. Additional information crucial for gas sensing is electronic structure and charge transfer, which are most commonly obtained by photoemission spectroscopies. Finally, since the gas sensing mechanism is ruled by adsorption, the techniques that provide data about the desorbing species are utilized, such as thermogravimetric analysis and thermal desorption spectroscopy. The analysis of desorption experiments provides insight into the mechanisms of adsorption and decomposition of analytes as well as decomposition, hence the stability of the sensing material.

For studying sensing mechanisms, the most powerful experimental tools are in situ spectroscopies, such as in situ Raman spectroscopy, diffuse reflectance IR (DRIFT), high-pressure XPS (HPXPS), and in situ electron paramagnetic resonance (EPR) spectroscopy. They serve to reveal the adsorbates and surface species when the surface of the sensing material is exposed to the gases, as well as to trace chemical states of elements in the sensing material. The information obtained from these methods can be directly compared with DFT results. Given that the theoretical model is as close to the experimental one as possible, in situ adsorption studies give the same output data as simulations, i.e., preferential adsorption sites, reaction pathways, electronic charge transfer/redistribution, and change in the electronic structure upon adsorption. Thus, they should be considered as a methodological basis for studying sensing mechanisms when the full intertwining of theory and experiment is desired.

**Figure 4:**
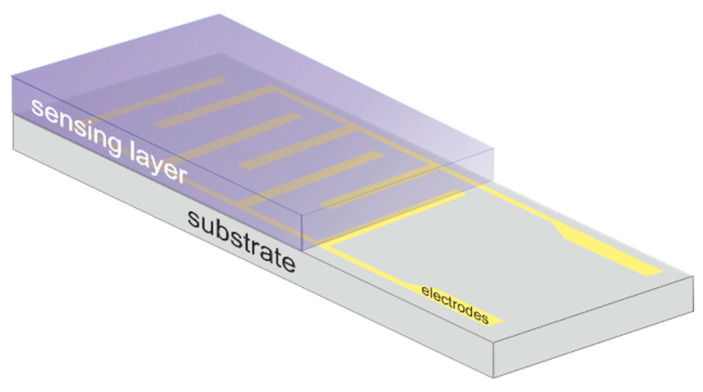
Schematic representation of sensor device construction.

The most common method for acquiring the sensor’s response to the analyte in the surrounding atmosphere is a simple measurement of changes in the electrical resistance of the sensing structure during the exposition to the gas. For this purpose, sensing materials are deposited on the substrate (e.g., glass or silicon) with interdigital electrodes (most often made of Au or Pt). Figure 4 shows the scheme of the most common sensor device. The device is placed in a chamber where the analyte can be provided at a given concentration in the carrier gas (air or nitrogen). The temperature and humidity should be controlled during experiments to establish their influence on the sensor’s response. The electrical feed-through allows real-time monitoring of the sensor’s electrical resistance. Sensor response is defined as a relative change in the resistance during the gas exposition compared to the resistance during pure carrier gas flow. The parameters that are tested are usually sensitivity, limit of detection, selectivity, optimum working temperature, and short-term and long-term stability.

## 5. Exemplary Works Joining Theory and Experiment

In Section 4 and Section 5, the theoretical and experimental methods applied in gas sensors studies have been presented, emphasizing the information useful in the gas sensing mechanism that can be obtained. However, to achieve the comprehensive mechanism description necessary for the effective device design, the theoretical and experimental data should be compared and put together in a way to provide a complete picture of the analyte-sensing material interaction and its possible implications for gas sensor construction. This is a challenging task since on the one hand the computational methods need to be optimized to achieve sufficient accuracy and on the other hand several advanced surface experiments on the actual sensing structure are required. The comparison of the results from both groups of methods is not always straightforward, because the theoretical systems typically need to be much less complex than the experimental ones for the sake of computational costs. Hence, the proper simplifications need to be assumed carefully in a way to gain the most realistic picture that is possible within the applied resources. Despite all of these obstacles, there have been a large number of successful research reports published in the last several years. These show that intertwining DFT study with experiments investigating gas sensing mechanisms is a promising direction to improve this field of science. This section provides some examples of those studies. A few studies are described in detail. The descriptions end with a summary from the point of view of intertwining the theory and experiment to show the strengths and weaknesses of the different approaches. Table 4 summarizes several other works in which joint theoretical and experimental studies were performed and succeeded in revealing the sensing mechanism.

**Figure 5:**
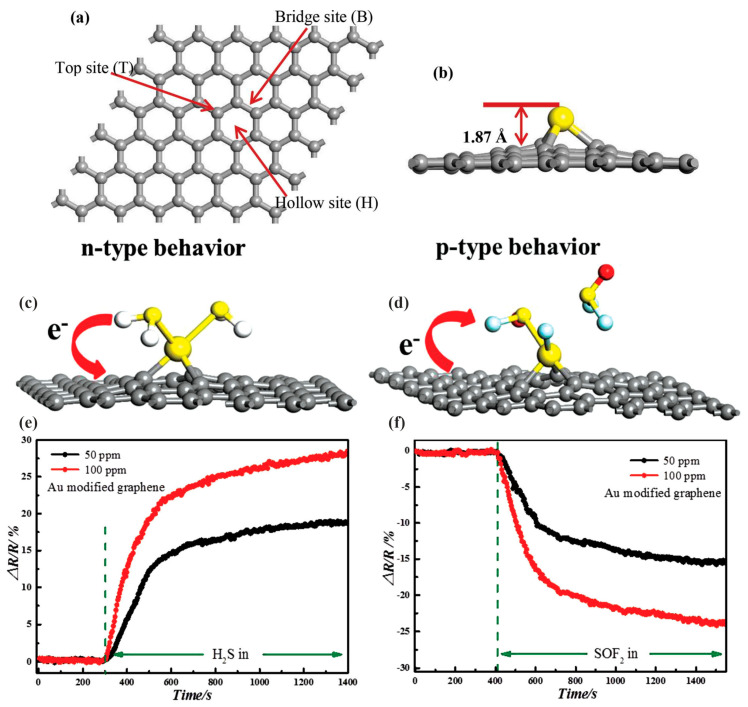
The summary of the most important outcomes of DFT investigation of H_2_S and SO_2_F adsorption on Au-modified graphene and experimental sensor responses. (**a**) Three possible modification positions of Au atom on graphene. (**b**) Side view of partial atomic structure for T site modification mode. (**c**) Theoretical adsorption geometry and corresponding charge transfer for H_2_S on Au-modified graphene. (**d**) Theoretical adsorption geometry and corresponding charge transfer for SO_2_F on Au-modified graphene. (**e**) Experimental sensor response of Au-modified graphene to H_2_S. (**f**) Experimental sensor response of Au-modified graphene to SO_2_F. Green dashed line indicates the time of analyte introduction. Reproduced from <Xiaoxing Zhang, Lei Yu, Xiaoquing Wu, Weihua Hu, Experimental Sensing and Density Functional Theory Study of H_2_S and SOF_2_ Adsorption on Au-Modified Graphene, *Adv. Sci.* **2015**, *2*, 1500101> in the compliance with CC BY 4.0 license [40].

**Figure 6:**
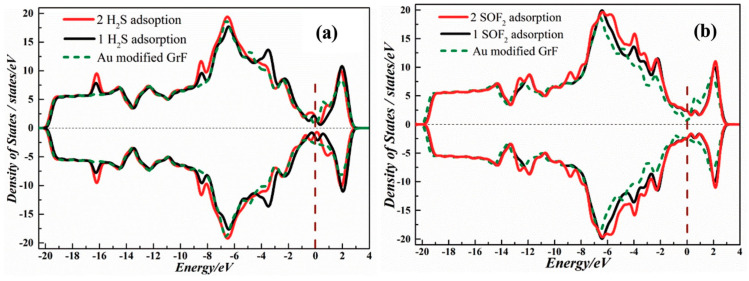
(**a**) Spin-polarized DOS of pristine graphene and Au-modified graphene, Au-modified graphene–H_2_S, and Au-modified graphene–2H_2_S. (**b**) Spin-polarized DOS of pristine graphene and Au-modified graphene, Au-modified graphene–SOF_3_, and Au-modified graphene–2SOF_3_. Reproduced from <Xiaoxing Zhang, Lei Yu, Xiaoquing Wu, Weihua Hu, Experimental Sensing and Density Functional Theory Study of H_2_S and SOF_2_ Adsorption on Au-Modified Graphene, *Adv. Sci.* **2015**, *2*, 1500101> in the compliance with CC BY 4.0 license [40].

Xiaoxing Zhang et al. [40] studied the adsorption of H_2_S and SOF_6_ on Au-modified graphene. The aim of the proposed sensor was application in detecting the insulation faults in power equipment since H_2_S and SOF_6_ are two characteristic products of SF_6_ decomposition. The investigation combined DFT calculations with several experimental techniques, such as XRD, Raman spectroscopy, scanning electron microscopy (SEM), transmission electron microscopy (TEM), high-resolution TEM (HRTEM), X-ray photoelectron spectroscopy (XPS), and electrical measurements of sensor responses. Among the experimental studies, spectroscopies and microscopies served mostly as tools for sensing material characterization before gas exposition. Sensing films of pure reduced graphene oxide (rGO) and Au-modified graphene were fabricated by applying layer-by-layer (LBL) deposition on copper IDEs etched on epoxy resin. For testing sensing properties, target gases (H_2_S and SOF_2_) diluted with pure and dry helium were delivered to sensors through a mass flow-controller. The electrical responses to two concentrations (50 ppm and 100 ppm) of both gases were measured several times in controlled conditions (room temperature and humidity of dry carrier gas). The results showed that rGO responds to H_2_S, but is not sensitive to SOF_2_. Au-modified graphene detects both gases. However, H_2_S causes a reduction in both materials’ electrical resistance, while for SOF_2_ and Au-modified graphene an increase in resistance is observed. For H_2_S, the sensitivity of Au-modified graphene is higher than that of pure rGO. Further investigation proves that the Au-modified graphene response is faster than that for pure rGO. To explain the sensing mechanism, a DFT study was performed. First, the model of Au-modified graphene was chosen. Three different bonding sites of Au on a graphene sheet were considered based on the experimental material’s characterization (Figure 5a). The geometry optimization was performed for each of them and one bonding site with the lowest formation energy (Figure 5b) was chosen for further analysis. The charge analysis shows electron depletion from Au atoms bonded to C atoms, which is with accordance with XPS measurements that revealed positively charged Au (I) and Au (III) ions. The calculated DOS spectrum indicates the change in conductance by introducing Au atoms. The presence of Au endows the material with metallic properties. For gases adsorption, different adsorption geometries were considered for single and double H_2_S and SOF_2_ molecules on Au-modified graphene. For every adsorption system, DOS spectra were also computed (Figure 6). The spectra showed the difference in DOS near Fermi level between pure Au-modified graphene and Au-modified graphene with adsorbed H_2_S. The surrounding DOS increases after the interaction. The Fermi level of Au-modified graphene shifts upward by 0.043 eV as a result of an n-type effect caused by H_2_S. In the case of SOF_2_, the effect is opposite, indicating a *p*-type effect. Moreover, the shift is much higher than in the case of H_2_S (1.05 eV). The theoretical results are mostly consistent with experimental sensing responses. They prove the chemisorption of both gases on Au-modified graphene and the charge transfer analysis explains the experimental directions of resistance changes (Figure 5). However, there is some discrepancy between theoretical and experimental results for H_2_S adsorption on pure graphene. The simulations show physisorption without charge transfer, while relatively high changes in measured electrical resistance would rather suggest chemical interaction and charge transfer. As a possible explanation, the authors postulate the heteroatoms, such as N and O harbored during the preparation of graphene films. Their presence was confirmed by XPS and Raman characterization. This issue points out that the theoretical and experimental results have to be always compared carefully, since in the experimental system there are many factors not included in simplified models that can significantly influence results.

Lili Wang et al. [2] explored the sensing mechanism in hybrid nanofiber-based room-temperature CO sensors. The material used for gas detection in this study was a one-dimensional (1D) hybrid nanofiber (HNF) decorated with ultrafine NiO nanoparticles (NiO NPS). This structure showed a highly selective response to CO low concentration (1 ppm) with very fast response and recovery time and high sensitivity. The experimental and DFT investigation provided insight into the mechanism of this excellent selective response. The NFs of TiO_2_ were synthesized by the electrospinning method. Next, the NiO NPs were prepared and 500 mm of NiO/TiO_2_ layer was spin-coated onto an Al_2_O_3_ substrate with Pt IDEs. The sample was characterized by field-emission scanning electron microscopy (FESEM), XRD, TEM, HRTEM, selected area electron diffraction (SAED), and BET analysis. Sensor responses were measured by monitoring the device’s electrical resistance. The pre-gas exposition characterization showed a very well-developed surface with many adsorption sites available, i.e., reactive NiO (110 facets), and an abundant interface that improved the charge transfer properties. The DFT calculations were performed before the gas exposition of the actual sample, mostly to verify which analyte interacts the strongest with the NiO (110) facets. The results of the theoretical study showed possible high selectivity toward CO. Measurements of the electrical sensing properties proved that the fabricated device is highly sensitive and selective toward CO, possesses a low detection limit (1 ppm), and exhibits very fast response and recovery times and reproducibility at room temperature. Afterward, characterization of the sensing experiment was performed to explain the sensing mechanism. TEM images with the corresponding EDS spectra were taken, contact potential measurements (CPD) were performed to obtain information about work function, and XPS spectra were recorded to analyze the presence of Ni in NiO/TiO_2_ HNFs. Results of experiments showed that the sensing mechanism is based on the depletion layer formation on the surface of the TiO_2_ NFs, and TiO2/NiO interface, caused by oxygen ion adsorption. The CO molecules react with adsorbed oxygen species or adsorb directly onto the NiO/TiO_2_ surface, which cause charge transfer and changes in electrical conductivity. The adsorption is additionally enhanced due to the highly exposed (110) NiO reactive facets that serve as active sites for oxygen species adsorption. In summary, in this work, a comprehensive experimental study was performed, while DFT calculations served as a tool for pre-selection of the analyte.

**Figure 7:**
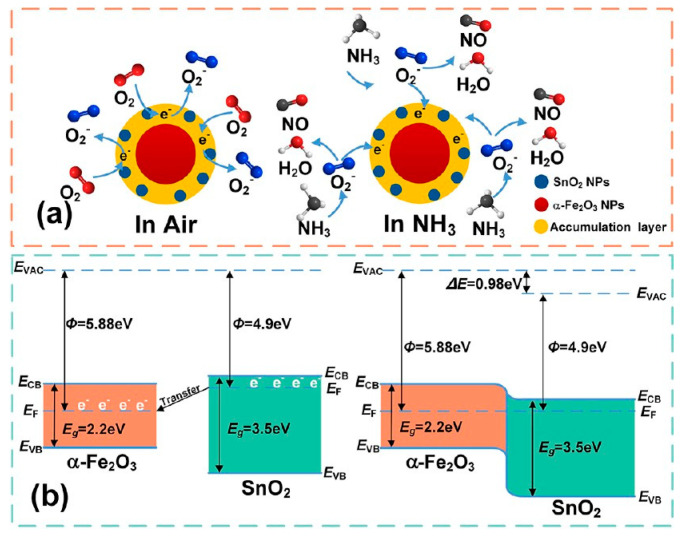
Sensing mechanism for NH_3_ interaction with tiny amorphous SnO_2_ nanoparticles modified on a-Fe_2_O_3_, concluded from the experimental techniques and literature review. (**a**) The gas adsorption process illustration; (**b**) Changes in the electronic structure caused by interaction with gas. A Schottky barrier on the Fe_2_O_3_ and SnO_2_ interface is formed, promoting electron accumulation in that region and improving the gas adsorption. There is also chemical sensitization since SnO_2_ nanoparticles act as catalysts for the chemical interaction of Fe_2_O_3_ with ammonia. Reproduced from [27] with permission from ACS Publications.

Lijia Xu et al. [27], starting from an idea of lowering the operating temperature of a-Fe_2_O_3_ by adding small metal oxide particles with highly exposed active sites, conducted a study of tiny amorphous SnO_2_ nanoparticle modification on a-Fe_2_O_3_ for room-temperature ammonia sensing. Similar to the works described above, the investigation consisted of the material’s surface characterization, experimental sensing properties measurements, and DFT calculations. The samples were prepared by the solvothermal method followed by suction filtration and drop coating on a ceramic tube with Au electrodes. The morphology of obtained composites was studied by TEM and SEM. It was shown that the addition of the Sn component introduces a porous surface and contributes to particle fusion. Simultaneously, a significant number of pores appear on the surface, potentially increasing the specific surface area. This was confirmed by BET analysis. XRD patterns and HRTEM images were obtained to identify the exposed crystal facets. These showed very few lattice stripes from SnO_2_, proving low content of Sn and poor SnO_2_ crystallinity. Chemical composition was analyzed by EDS and XPS. The most important result from EDS was the even distribution of Sn around the particles, and again, its low content compared to other elements. XPS spectra revealed the presence of oxygen vacancies that can improve gas sensitivity. The gas sensing performance experiment showed improved response time and sensitivity to ammonia at room temperature for a-Fe_2_O_3_@SnO_2_ sensor, compared to pure a-Fe_2_O_3_. The sensing mechanism was first concluded from the experimental techniques and literature review and is summarized in Figure 7. Typically, for sensors based on metal oxides, oxygen pre-adsorbed on the surface plays the main role in the interaction with the analyte and consequent charge transfer. In the case of the studied composite, a Schottky barrier on the Fe_2_O_3_ and SnO_2_ interface is formed, promoting electron accumulation in that region and improving the gas adsorption. There is also chemical sensitization since SnO_2_ nanoparticles act as catalysts for the chemical interaction of Fe_2_O_3_ with ammonia. Furthermore, oxygen vacancies ease the molecules’ diffusion and interaction with the materials, improving response time. The DFT study to some extent confirmed the described sensing mechanism. Computed adsorption energies for the most stable adsorption configuration of ammonia on pure Fe_2_O_3_ (110) and Fe_2_O_3_ (110) surfaces with small SnO_2_ crystallites proved that the studied analyte interacts more strongly with SnO_2_-decorated Fe_2_O_3_ than with the pure Fe_2_O_3_ surface. The electrical sensitization was verified by calculating the changes in DOS spectra after gas adsorption. The results showed some hybridization in the spin-up channel, resulting in energy band alignment when the two composite materials come into contact. Also, the hybridization of Fe and N is significantly enhanced. However, the theoretical investigation only supported the experimental study in some aspects but did not provide the full image of the sensing mechanism.

**Figure 8:**
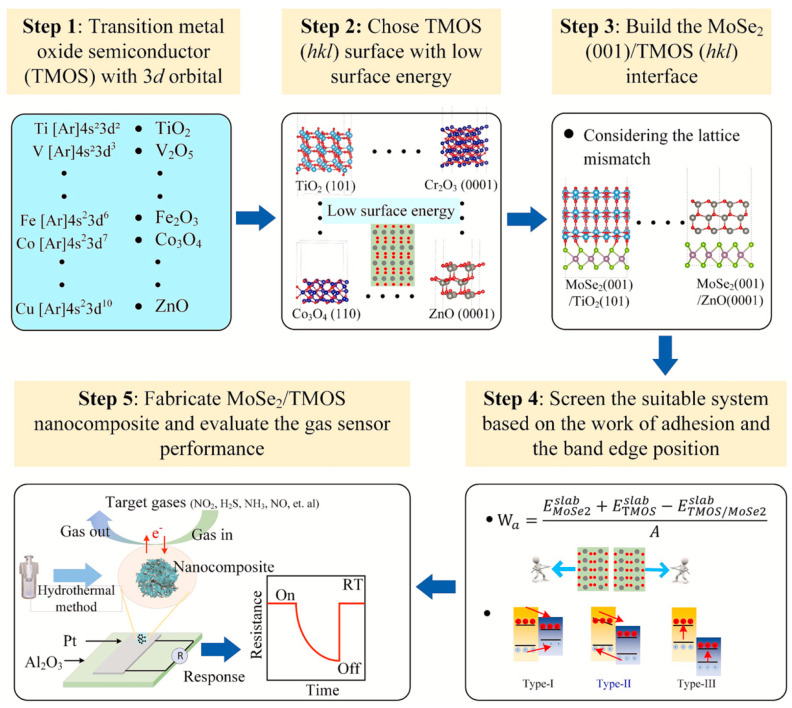
The schematic diagram of the screening process for the optimum MoSe_2_/3d-TMOS heterostructure for gas sensing. Reproduced from [50] with permission from Elsevier.

Xiangzhao Zhang et al. [50] proposed a different approach to the gas sensor design than the works described above. The computational screening to choose the optimum transition metal oxide with 3d orbital (3d-TMOS) for the heterojunction with MoSe_2_ was performed before the experimental fabrication of the device. The choice of 3d-TOS was based on the work of adhesion and the positions of band edges with reference to MoSe_2_. The suitable band edge positions can significantly improve charge transfer. The fast charge separation contributes to the resistance change rate, increasing sensor response. For the screening procedure, the following 3d-TMOS were chosen: TiO_2_, V_2_O_5_, Cr_2_O_3_, MnO_2_, Fe_2_O_3_, Co_3_O_4_, NiO, and ZnO. For MoSe_2_, the (001) surface was considered. The surface of each TMOS was chosen based on calculated surface energies and the smallest lattice mismatch between TMOS and MnSe_2_. For each heterostructure, the interfacial binding strength and energy levels were calculated. The schematic diagram of the screening process is presented in Figure 8. Among the oxides studied, Co_3_O_4_ and Fe_2_O_3_ revealed promising properties for gas sensors. Thus, MoSe_2_/Co_3_O_4_ heterostructures with different Co_3_O_4_ ratios with respect to MoSe_2_ were prepared for the experimental investigation. The as-fabricated materials were analyzed by XRD, XPS, TEMS, HRTEM, BET, and UV-Vis spectroscopy. Gas sensing devices were prepared by drip-casting materials on platinum electrodes and their performance to various target gases was evaluated. The phase and microstructure analysis showed mostly a high purity for the fabricated heterostructures. The gas sensing performance evaluation was performed for NO_2_ detection at room temperature. To study the influence of Co_3_O_4_ loading on sensing performance and to select the optimum one, samples with different MoSe_2_/Co_3_O_4_ ratios were exposed to the target gas. These tests confirmed improved parameters such as sensitivity, selectivity, response and recovery times, and stability of MoSe_2_/Co_3_O_4_ compared to pure MoSe_2_. To further evaluate the sensing mechanism, DFT calculations were again employed. Particularly, adsorption energies and charge distribution were computed. The improved sensing performance after the formation of the MoSe_2_/Co_3_O_4_ structure, based on the comprehensive analysis of theoretical and experimental results, was ascribed to the formation of the type-II heterojunction, leading to the fast charge extraction rate in the MoSe_2_/Co_3_O_4_ nanocomposite. The comparison of theoretical and experimental results, as in most other works, helped to describe the most likely gas sensing mechanism. However, the extended DFT part concerning the screening before the actual sensor preparation is a less common approach that should be considered in the works for which sufficient computational resources are available.

One of the emerging groups of materials studied recently for gas sensing is MXenes. However, according to Guoqing Feng et. al. [48], several critical issues must be overcome to obtain effective sensing devices based on these 2D materials. They point out such constraints as weak gas adsorption capacity, poor sensitivity, and limited selectivity. Thus, they propose quantum dots (QDs)-sensitization of few-layered Ti_3_C_2_T_x_ for ultrasensitive trace NO_2_ detection. The study comprises 2D/0D MXene/PbS heterostructures synthesis (the series of samples with different PbS concentrations), microstructure characterization, gas sensing performance tests, and DFT calculations of 37 models. XRD, XPS, SEM, EDS, HRTEM, and BET techniques were applied for the experimental part. The sensing responses for NO_2_ and several interfering analytes were measured at room temperature and under various relative humidity levels.

The measured XRD patterns, XPS spectra, and microscopic images with corresponding structural and chemical analysis confirmed the successful preparation of heterostructures of 2D MXene with QDs randomly dispersed or aggregately anchored onto the surface of few-layered MXene. BET analysis revealed that MXene provides the adsorption area for QDs anchoring, and stacked QDs contribute to the formation of mesopores beneficial to enhance gas adsorption. Gas sensing tests showed improved sensing performance toward NO_2_ of heterostructures compared to the simple few-layered MXene. Moreover, the sensitivity can be tuned by choosing the proper PbS concentration.

**Figure 9:**
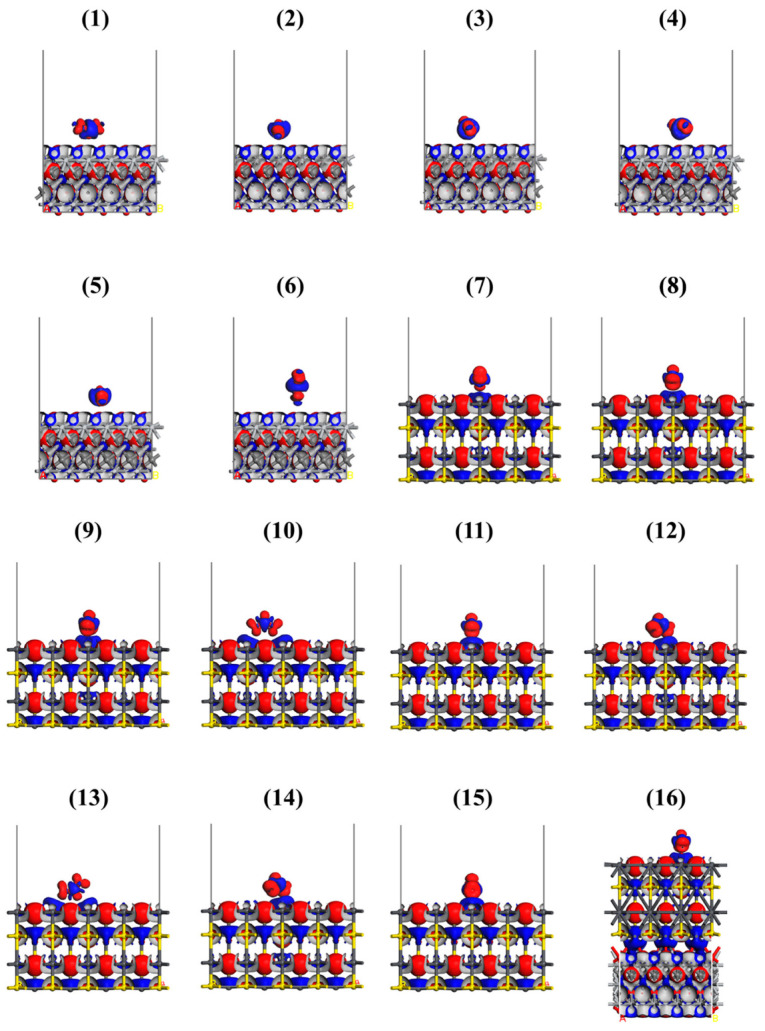
The adsorption geometries of NO_2_ on MXene and corresponding electric charge distributions. The red represents electron accumulation and blue shows electron depletion. A total electron depletion area occurs between the MXene surface and O atoms. The region around the N atom of NO_2_ exhibits a smaller blue area than red, confirming the electron-withdrawing property of NO_2._ Reproduced from [48] with permission from Elsevier.

The DFT calculations of adsorption energies and charge transfer were performed for NO_2_ and O_2_ interaction with MXene and PbS. Various adsorption configurations were taken into account (Figure 9). As a computational model of the MXene studied, O-terminated Ti_3_C_2_ (Ti_3_C_2_O_2_) was chosen as the most energetically stable structure based on the previous works. The computed adsorption energies confirmed theoretical findings: i.e., the higher sensitivity of heterostructure compared to the separate components occurred due to the strong chemisorption of NO_2_ on the PbS. The charge analysis showed that the potential electrical conductivity variations were due to the adsorption. Moreover, the optimized model of heterostructures revealed the formation of lead–oxygen bonds, explaining the appearance of a satellite peak in the experimental XPS spectrum.

**Figure 10:**
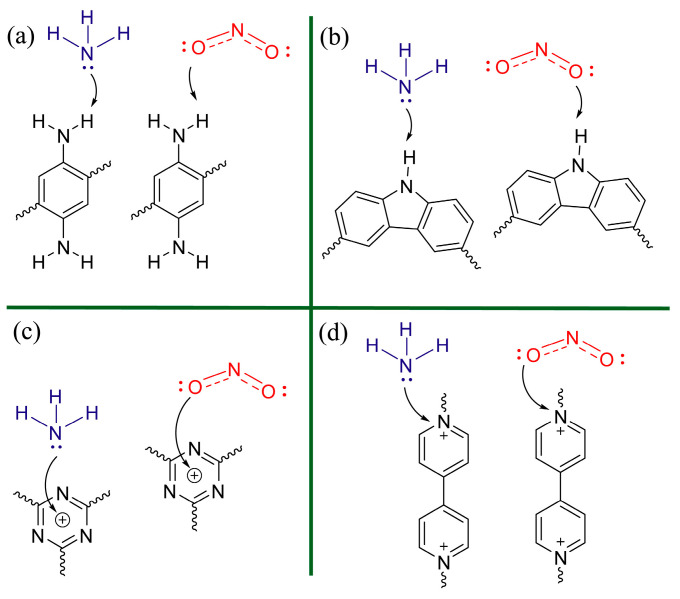
Proposed hydrogen bonding and coordination interaction of NH_3_ or NO_2_ with (**a**) primary amine, (**b**) secondary amine, (**c**) triazine ring, and (**d**) viologen group. Reproduced from [16] with permission from Elsevier.

Recent gas sensor research is not only focusing on inorganic materials. Many organic compounds have been investigated as a potential base for the design of sensing devices. Jia-Lin Zhu et. al. [16] explored in their work the performance of various nitrogen-contained covalent organic polymers and frameworks in ammonia and nitrogen dioxide detection. They chose these compounds since N-contained groups are important adsorption sites for target molecules. Thus, they designed four N-contained groups in different configurations (i.e., primary amine, secondary amine, tertiary amine, and quaternary ammonium) to verify the configuration on sensing properties. Similar to the research described above, this study consisted of the following steps: material synthesis and sensor preparation, material characterization, sensing properties testing, and sensing mechanisms evaluation based on DFT computations and experimental results. The characterization was performed by techniques such as XPS, IR spectroscopy, and XRD. Results confirmed successful synthesis of the designed materials. For the gas sensing performance tests, thin layers of the materials were deposited on Au IDEs. The sensing responses to twelve different harmful gases were measured at room temperature. The results revealed very high sensitivity of primary amine, secondary amine, and tertiary amine to NH_3_, while quaternary ammonium was mainly adsorbing NO_2_. The interaction mechanisms were investigated by applying DFT adsorption calculations together with experimental in situ UV-Vis spectroscopy. From the DFT, the detailed information about preferred adsorption geometries, adsorption energies, and charge distribution were obtained. UV-Vis spectroscopy was performed for the materials without target analytes and during NH_3_ or NO_2_ exposition. Computations confirmed that primary amine, secondary amine, and tertiary amine interacted with NH_3_ through hydrogen bonding, while NO_2_ interacted through coordination with quaternary amine nitrogen atoms. The UV-Vis experiments further confirmed the exact active sites for NH_3_ and NO_2_ (Figure 10).

## 6. Summary and Outlook

This review showed enormous progress has been made in investigating sensing mechanisms for the purpose of designing more efficient gas-detecting devices, especially from the point of view of joining theoretical and experimental approaches in one productive workflow. Our aim was to emphasize the significance of a comprehensive approach that leads to a more efficient utilization of gas adsorption influence on material properties in improving the performance of gas sensors. Currently, it is especially important because of the growing need for precise monitoring of various chemical substances in the atmosphere, for human health and safety and environmental protection. We showed that computational quantum chemistry and experimental materials science provide highly effective tools for the description of gas sensing mechanisms, especially when theory and experiment come together and complement each other. Fortunately, researchers in the field noticed this powerful potential; in the last several years, the number of studies regarding gas sensors that join theory and experiment has grown significantly. The exemplary results presented show that this path leads to improvements in device design concerning aspects such as sensitivity, selectivity, working temperature, or response time. We believe that this field will continue to grow, especially from the side of computational studies and their correlation with actual experimental systems. Now, the greatest obstacle to overcome is choosing a model simple enough for the computational resources but, at the same time, representing as well as possible the experimental one. However, with growing computational power and the development of more efficient approximations for quantum chemistry methods, hopefully, these limitations will become less challenging in the future. Currently, for materials most commonly applied in gas sensors (i.e., SnO_2_, ZnO, graphene), there are many theoretical studies that can be utilized to find efficient computational techniques and input parameters. With this growing field, it will be possible to create databases for benchmarking the systems, which will facilitate the methodology. Another aspect is the proper interpretation of the results from both sides. Such an approach requires interdisciplinary collaboration of scientists whose background is in materials theory with experts in experimental characterization of material properties. The result is then a description of phenomena ruling the gas sensing mechanism. At the end, for the proper utilization of these mechanisms, engineers must design and test the device based on the outcome of the fundamental research. In the era of multidisciplinary science, this is an interesting area to explore. The best scenario is an application of methodology in which firstly the sensitive and selective device is designed on the theoretical level with moderate computational costs involved, and then experiments serve to verify the successful fabrication.

## Figures and Tables

**Table 2:** The XC functionals and basis set/pseudopotential combinations used for modeling gas sensing mechanisms, together with respective materials, applied software, and literature references.

XC Functional, Basis Set/Pseudopotential	Materials	Software	References
LDA and GGA PBE, norm-conserving Troullier–Martins (DFT + U)	CuO	SIESTA	[22]
GGA PBE-D, semicore pseudopods	Au-modified graphene	DMol^3^	[40]
B3LYP, 6-31 + G (d, p)	Porphyrinated polyimide honeycomb, covalent triazine-based frameworks	Gaussian 03, Gaussian 09	[14,15]
GGA, norm-conserving Troullier–Martins	graphene	CASTEP	[41]
B3LYP, 6-311 G (d, p)	squariaine	Gaussian 09	[37]
GGA PBE-D3, ultrasoft Vanderbilt’s (DFT + U)	ZnO	Quantum Espresso	[19]
GGA PBE, pseudopotential generated based on PAW	amine-functionalized ZIF-8 with 3-amino-1,2,4-triazole, tellurene nanoflake, a-MoO_3_, atomically dispersed metals, SnO_2_, Al_2_O_3_/ZnO, MoSe2/3*d*–TMOS	VASP	[4,13,17,25,38,50,51]
GGA PBE, DNP	SnO_2_, Ni-doped ZnO, SnO_2_ (110), Pd_4_ cluster decorated SnO_2_, ZnO/CuO	DMol^3^	[7,10,30,31,32]
B3LYP-gCP-D3, LANL2DZ and cc-pVDZ	ZnO nanotube, Pd-decorated ZnO nanocluster	GAMESS	[20,52]
GGA PBE, DSPP	Pd-doped ZnO	DMol^3^	[8]
GGA PBE-D3-(BJ),, pseudopotential generated based on PAW (DFT + U)	ZnO:Ag with AgAu and AgPt	VASP	[51]
GGA PBE	Zn-substituted SnS, covalent organic frameworks, SnO_2_/SnSe_2_	CASTEP	[34,39,44]
GGA PBEsol	Sr-Doped cubic In_2_O_3_/rhombohedral In_2_O_3_ homojunction	VASP	[9]
B3LYP-D3	Hematite rhombuses	CRYSTAL17	[49]
GGA PBE-D	Au-SnSe_2_, graphene	DMol^3^	[42,45]
B3LYP, 6-31 + G and LANL2DZ (DFT-D)	Ag-decorated ZnO graphene-like nanosheet	GAMESS	[53]
Norm-conserving pseudopotential within Troullier–Martin scheme	PEDOT:PSS	SIESTA	[36]
GGA PBE, pseudopotential generated based on PAW (DFT + U)	Ni-doped CeO_2,_ Co_3_O_4_(110) surface, SnO2 nanoparticle- modified *α*-Fe_2_O_3_	VASP	[5,27,29]
GGA PBE_D3, def2-SVP and def2-TZVP	Liquid crystals	Gaussian 09	[43]
GGA PBE-D3, ultrasoft pseudopotentials, (DFT + U)	ZnO nanorods	Quantum Espresso	[54]
GGA PBE, ultrasoft pseudopotentials	defect-enriched MoO_3_	VASP	[24]
GGA PBE-D3, pseudopotential generated based on PAW	MoS_2_/WS_2_ nanoflowers	VASP	[47]
GGA PBE-D3-(BJ), pseudopotential generated based on PAW	Al_2_O_3_/ZnO	VASP	[55]

**Table 3:** Experimental methods applied in evaluating gas sensing mechanisms.

Method	Information
X-ray diffraction (XRD)	Structural characterization of the sensing layer: the favorable crystal planes exposed for the interaction with adsorbate, crystallite size, degree of crystallization, lattice constants
Raman spectroscopy	Structural characterization of the sensing layer: molecular structure, purity
Nuclear magnetic resonance spectroscopy (NMR)	Structural characterization of the sensing layer: molecular structure, purity
Infrared spectroscopy	Structural characterization of the sensing layer: molecular structure, purity
Electron microscopies (SEM and TEM)	Morphological characterization of the sensing layer’s surface: active sites availability, surface roughness, grain size; Structural characterization of the sensing layer: crystallite size, degree of crystallization, lattice constants
Atomic force microscopy (AFM)	Morphological characterization of the sensing layer’s surface: active sites availability, surface roughness, grains size
Photoemission spectroscopies (XPS, UPS)	Structural/chemical characterization: chemical composition, purity, chemical bonds Electronic characterization: changes in electronic structure during the interaction with analytes, charge transfer
Thermogravimetric analysis (TGA)	Temperature and products of thermal degradation of the sensing layer: temperature of analyte desorption
Thermal desorption spectroscopy (TDS)	Temperature and products of thermal degradation of the sensing layer: temperature of analyte desorption, desorption energies
Brunauer–Emmett–Teller (BET) analysis	Pore volume and surface area (adsorption site availability)
In situ spectroscopies	Adsorbates and surface species: chemical states of elements in sensing material

**Table 4:** Summary of joint theoretical–experimental works revealing sensing mechanisms.

**Sensing Material**	**Target Gas**	**Calculation Method**	**Calculated Structure**	**Results of Calculation**	**Experimental Techniques**	**Experimental Results**	**Reference**
Ni-doped CeO_2_	H_2_S	GGA PBE, pseudopotential generated based on PAW (DFT + U)	Ni-doped (110) CeO_2_ surface with oxygen vacancy	H_2_S adsorption energy; doping effect of Ni in electron transfer—V_O_ enhancement	XRD, FESEM, HRTEM, XPS, DRIFTS, Raman spectroscopy, BET, DRS, PLS, SR	(111), (220), (311) CeO_2_ facets; morphology change and lattice contraction upon Ni doping; mesoporous structure; enhancement of V_O_ with Ni doping, the H_2_S adsorption pathway	[5]
Pd-doped ZnO	aniline	GGA PBE, DSPP	Pd-doped/undoped (100) ZnO surface −72 atoms supercell	Adsorption energy of aniline on undoped and Pd-doped ZnO (−0.87 eV and −1.93 eV); band gap alteration with Pd doping and aniline adsorption	XRD, FESEM, TEM, XPS, BET, UV-Vis, SR	ZnO hexagonal facets c-axis suppression by Pd doping; porous structure; pre-absorbed O species number grows after Pd doping; low Pd introduces electron depletion layer in ZnO, excessive Pd accumulation reduces surface adsorption sites availability	[8]
Sr-doped cubic In_2_O_3_/rhombohedral In_2_O_3_ homojunction	ethanol	GGA PBEsol	Pristine and Sr-doped cubic In_2_O_3_ (222) surface, and rhombohedral In_2_O_3_ (110) surface	Ethanol adsorption energy on pristine and Sr-doped surfaces on different adsorption sites; differential cohesive energy—Sr doping may cause phase change from cubic to rhombohedral	SEM, TEM, EDS, XRD, XPS, Raman spectroscopy, SR	Polycrystalline structure; Sr distribution not uniform; phase transition from cubic to rhombohedral upon Sr doping; electron depletion layer on homojunction between two phases	[9]
Tellurene	NO_2_	GGA PBE, pseudopotential generated based on PAW	Tellurene sheet	Adsorption energies and charge transfer for various adsorption configurations of CO_2_, N_2_, NO_2_, and O_2_; band structures and PDOS before and after gas adsorption	TEM, AFM, Raman spectroscopy, SR	Ambient oxygen species spontaneously adsorbed on tellurene surface	[38]
Covalent organic frameworks	NO_2_	GGA PBE	Molecule of squaraine (SA) with 1,3,5-tris(4-aminophenyl)benzene (TAPB)	Adsorption energies and charge transfer for various adsorption configurations of NO_2_	HRTEM, EDS, XRD, XPS, BET, TGA, SR	AA stacking with hexagonal structure; mesoporous properties; low BET surface;	[39]
ZnO nanorods	ethanol	GGA PBE-D3, ultrasoft pseudopotentials (DFT + U)	(10-10) surface of orthorhombic ZnO structure	Influence of hypothetic species present in the reaction pathway on the electronic structure	XRD, SEM, HAADF-STEM, BET, DRIFTS	Reaction pathway for ethanol adsorption	[55]
Oxygen-vacancy-enriched a-MoO_3_	trimethylamine	GGA PBE, pseudopotential generated based on PAW	α-MoO3-400, the (110) plane of the perfect crystal of α-MoO3 and the α-MoO3 containing O vacancy (Ov-α-MoO3)	Adsorption energies and charge transfer; O vacancy influence on electronic structure	XRD, SEM, TEM, FT-IR, XPS, EPR, BET, SR	High purity of α-MoO3 nanosheets; influence of calcination temperature on pore size; O vacancies appearance	[25]
CuO	O_3_	LDA and GGA PBE, norm-conserving Troullier–Martins (DFT + U)	(111) CuO surface (64 atoms)	Point-defects formation energies; oxygen vacancies influence on electronic structure; adsorption energy of O_3_ and charge transfer	XRD, SR	(111) plane favored	[22]
